# Patient outcomes and return to play after arthroscopic rotator cuff repair in overhead athletes: a systematic review

**DOI:** 10.1186/s10195-023-00683-w

**Published:** 2023-01-19

**Authors:** Filippo Migliorini, Giovanni Asparago, Francesco Cuozzo, Francesco Oliva, Frank Hildebrand, Nicola Maffulli

**Affiliations:** 1 Department of Orthopaedic, Trauma, and Reconstructive Surgery, RWTH University Hospital, Pauwelsstraße 30, 52074 Aachen, Germany; 2 Department of Medicine, Surgery and Dentistry, University of Salerno, 84081 Baronissi, SA Italy; 3 Faculty of Medicine, School of Pharmacy and Bioengineering, Keele University, ST4 7QB Stoke On Trent, England; 4 Queen Mary University of London, Barts and the London School of Medicine and Dentistry, Centre for Sports and Exercise Medicine, Mile End Hospital, E1 4DG London, England

**Keywords:** Rotator cuff, Shoulder, Athletes, Arthroscopy, Repair, Overhead, Return, Treatment, Outcomes, Complication

## Abstract

**Background:**

Rotator cuff tear injuries in overhead athletes are common and may lead to chronic pain and joint disability, impairing sport participation and leading to premature retirement. The improvement of the patient reported outcome measures (PROMs) was evaluated, as were the time and level of return to sport and the rate of complication in overhead athletes who had undergone arthroscopic rotator cuff repair.

**Methods:**

This systematic review was conducted according to the Preferred Reporting Items for Systematic Reviews and Meta-Analyses: the 2020 PRISMA statement. In September 2022, the following databases were accessed: Pubmed, Web of Science, Google Scholar and Embase. No time constraints were used for the search. All the clinical trials investigating arthroscopic rotator cuff repair in overhead athletes were accessed.

**Results:**

Data from 20 studies were collected. The mean length of the follow-up was 40 months. All PROMs improved at last follow-up: Kerlan-Jobe Orthopaedic Clinic score (*P* = 0.02), visual analogue scale (*P* = 0.003), Constant score (*P* < 0.0001), University of California Los Angeles Shoulder score (*P* = 0.006) and American Shoulder and Elbow Surgeons’ score (*P* < 0.0001). Elevation also improved (*P* = 0.004). No difference was found in external and internal rotation (*P* = 0.2 and *P* = 0.3, respectively). In total, 75.4% (522 of 692 of patients) were able to return to play within a mean of 6.4  ±  6.0 months. Of 692 patients, 433 (62.5%) were able to return to sport at pre-injury level. Fourteen out of 138 patients (10.1%) underwent a further reoperation. The overall rate of complications was 7.1% (20 of 280).

**Conclusion:**

Arthroscopic reconstruction of the rotator cuff is effective in improving function of the shoulder in overhead athletes, with a rate of return to sport in 75.4% of patients within an average of 6.4 months.

**Level of evidence:**

III, systematic review.

*Trial registration* : Not applicable.

## Introduction

Shoulder injuries in overhead athletes are common and may lead to chronic pain and joint disability [1, 2]. Among shoulder injuries, rotator cuff tears are common [3, 4]. Rotator cuff injuries in overhead athletes may impair sport participation and lead to premature retirement [5–7]**.** The management of rotator cuff injuries in overhead athletes may be challenging [5, 8, 9]**.** Conservative management, such as injection therapy, non-steroidal anti-inflammatory medications, or physiotherapy, although able to reduce symptoms, often lead to poor functional outcome [10–13]**.** When conservative management fails, surgical management may be considered [14–16], and arthroscopy can improve shoulder function and achieve fast return to sport [17]. Current evidence on the efficacy and safety of arthroscopic rotator cuff repair in overhead athletes is limited, and a comprehensive systematic review of the literature is missing; therefore, a systematic review was conducted. The improvement of the patient reported outcome measures (PROMs) from baseline to last follow-up was evaluated, as were the time and level of return to sport and the rate of complication in overhead athletes who had undergone arthroscopic rotator cuff repair.

## Methods

### Eligibility criteria

All the clinical trials investigating arthroscopic rotator cuff repair in overhead athletes were accessed. Only studies published in peer reviewed journals were considered for inclusion. Given the authors’ language capabilities, articles in English, German, Italian, French and Spanish were eligible. Studies with level I to IV of evidence, according to Oxford Centre of Evidence-Based Medicine [18], were considered. Reviews, opinions, letters, and editorials were not considered. Animals, in vitro, biomechanics, computational, and cadaveric studies were not eligible. Data from national registries were not considered. Studies that reported the outcomes of open rotator cuff repair were not eligible. Studies that reported data on athletes from all leagues were suitable. Only articles reporting a minimum follow-up of 18 months were included. Only articles reporting quantitative data under the outcomes of interest were considered for inclusion. Eligibility criteria are shown in Table 1.

**Table 1:** Eligibility criteria

Inclusion criteria	Exclusion criteria
Clinical trials investigating arthroscopic rotator cuff repair in overhead athletes	Reviews, opinions, letters, editorials
Studies published in peer reviewed journals	Animals, in vitro, biomechanics, computational and cadaveric studies
Studies on athletes from all leagues	Data from national registries
Articles with minimum follow-up of 18 months	Studies of open rotator cuff repair
Articles reporting quantitative data	

### Search strategy

This systematic review was conducted according to the Preferred Reporting Items for Systematic Reviews and Meta-Analyses: the 2020 PRISMA statement [19]. The PICOT algorithm was preliminarily set with:

- P (Population): Overhead athletes;
- I (Intervention): Arthroscopic rotator cuff repair;
- C (Comparison): clinical outcomes;
- O (Outcomes): PROMs and rate of complications.
- T (Timing): 24 months’ follow-up.

In September 2022, the following databases were accessed: Pubmed, Web of Science, Google Scholar and Embase. No time constraints were used for the search. The following keywords were used in combination using the Boolean operators AND/OR: *shoulder, rotator cuff, repair, arthroscopy, athletes, sport, activity, overhead, ball, patient reported outcome measures, PROMs, range of motion, ROM, elevation, rotation, complications, revision, return to sport*.

### Selection and data collection

Two authors (FM; GA) independently performed the database search. All the resulting titles were screened and, if suitable, the abstract was accessed. The full-text of the abstracts that matched the topic were accessed. The bibliographies of the full-text articles were also screened by hand for inclusion. Disagreements were debated, and the final decision was made by a third author (NM).

### Data items

Two authors (FM;GA) independently performed data extraction. Generalities and demographics of the included studies were extracted: author and year of publication, journal, study design, number of athletes and shoulders enrolled in the study, mean duration of symptoms and follow-up, mean age, women. Data with regard to the following PROMs were collected at baseline and last follow-up: the Kerlan-Jobe Orthopaedic Clinic (KJOC) [20], 0–10 visual analogue scale (VAS), Constant score [21], University of California Los Angeles Shoulder score (UCLA-S) [22], American Shoulder and Elbow Surgeons’ (ASES) [23] and range of motion, ROM (shoulder elevation, external and internal rotations). The frequency with which patients returned to sport and the length of time it took, together with the relevant degree of activity, were also obtained. The rate of complications and revisions was recorded.

### Methodology quality assessment

The methodological quality assessment was performed through the Coleman Methodology Score (CMS), independently by two authors (FM;GA). The CMS is a reliable and validated tool for evaluating the methodological quality of systematic reviews and meta-analyses [24]. This score analyses the included articles evaluating the population size, length of follow-up, surgical approach, study design, description of diagnosis, surgical technique and rehabilitation. Additionally, outcome criteria assessment and the subject selection process were also evaluated. The quality of the studies scored between 0 (poor) and 100 (excellent), with values > 60 considered satisfactory.

### Synthesis methods

The statistical analyses were performed by the main author (FM) using IBM SPSS software version 25. For descriptive statistics, the mean and standard deviation were used for continuous data, while the percentage of events was used for binary data. To assess improvement from baseline to last follow-up, the mean difference effect measure was used. The *t*-test was performed with values of P < 0.05 considered statistically significant.

## Results

### Study selection

**Fig. 1:**
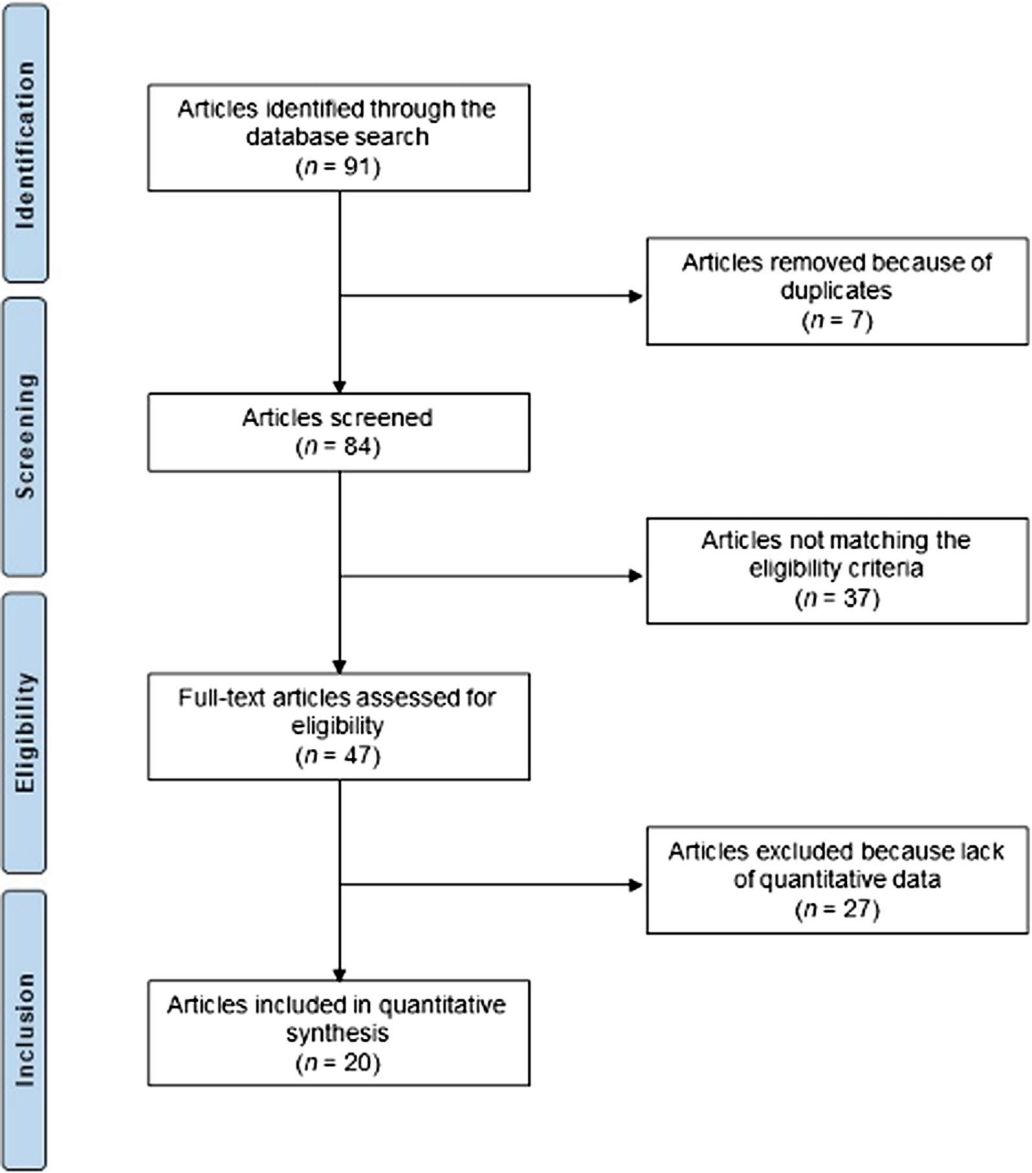
Flow chart of the literature search

The literature search resulted in 91 articles. After removal of duplicates (*N* = 7), a further 84 articles were not eligible for the following reasons: study design (*N* = 13), language limitation (*N* = 7), short follow-up (*N* = 17) and lacking quantitative data under the endpoint of interest (*N* = 27). Finally, 20 studies were included: four prospective, one case series, and 15 retrospective clinical studies. The literature search results are shown in Fig. 1.

### Methodology quality assessment

The CMS identified limitations and strengths of the present study. The study size and length of the follow-up were adequate. Surgical approach and diagnosis were well described by most articles.

Outcome measures and timing of assessment were frequently defined, providing moderate reliability. The procedures for assessing outcomes were often fair. Concluding, the mean CMS resulted in 64 points, attesting the fair quality of the methodological assessment. Details of the CMS of each article are shown in Table 2.

**Table 2:** Generalities and patient baseline of the included studies

Author et al., year	Journal	Design	CMS	Follow-up (*months*)	Patients (*n*)	Shoulders (*n*)	Mean age	Female (n)	Women (*%*)	Competitive athletes (n)
Anderson et al., 2006 [25]	*Am J Sports Med*	Prospective	83	30	48	52	58	18	37	39
Antoni et al., 2016 [26]	*Orthop Traumatol Surg Res*	Retrospective	67	45	76	76	57	37	49	76
Azzam et al., 2018 [7]	*Am J Sports Med*	Retrospective	53	74	32	32	16	4	13	32
Bhatia et al., 2015 [27]	*Am J Sports Med*	Retrospective	58	43	44	49	73	11	25	44
Boileau et al., 2009 [28]	*Am J Sports Med*	Prospective	62	35	10 (Suture Anchor)	10	37	0	0	
					15 (Interference Screw)	15	52	6	4	
Cohen et al., 2006 [29]	*Arthroscopy*	Retrospective	71	44	39	39	34	2	5	39
Ide et al., 2005 [30]	*Am J Sports Med*	Case series	65	41	40	40	24	7	17	30
Ide et al., 2005 [31]	*Am J Sports Med*	Prospective	73	39	17	17	42	3	18	6
Kim et al., 2002 [32]	*J Bone Joint Surg Am*	Retrospective	65	33	34	34	26	4	12	
Krishnan et al., 2008 [33]	*Arthroscopy*	Retrospective	65	26	23	23	37	8	35	3
Liem et al., 2008 [4]	*Am J Sports Med*	Prospective	68	26	21	21	59	7	33	21
Merolla et al., 2018 [34]	*Musculoskelet Surg*	Retrospective	66	24	38	38	27	21	55	38
Neri et al., 2011 [35]	*Am J Sports Med*	Retrospective	71	38	23	23	25	0	0	23
Park et al., 2013 [36]	*Am J Sports Med*	Retrospective	64	46	24	24	23	6	25	
Peduzzi et al., 2019 [37]	*Orthop Traumatol Surg Res*	Retrospective	71	95	135	135	29	32	24	112
Simon et al., 2017 [38]	*Wilderness Environ Med*	Retrospective	51	27	12	12	55	2	17	12
Spencer et al., 2010 [39]	*Clin Orthop Relat Res*	Retrospective	65	29	20	20	41	4	20	2
Van Kleunen et al., 2012 [40]	*Am J Sports Med*	Retrospective	45	37	17	17	19	0	0	17
Young et al., 2017 [41]	*Clin J Sport Med*	Retrospective	53	39	8	8	24	8	100	8
Yung et al., 2008 [42]	*Knee Surg Sports Traumatol Arthrosc*	Retrospective	61	28	16	16	24	3	19	5

*CMS* Coleman Methodology Score

### Study characteristics and results of individual studies

Data from 692 patients (701 shoulders) were collected. One hundred and eighty-three out of 692 patients (26.4%) were women. Five hundred and seven of 692 patients (73.3%) were athletes involved in competitions. The mean duration of symptoms was 10.8 ± 7.6 months, and the mean length of the follow-up was 40 ± 17.1 months. The mean age of the patients was 37.2 ± 16 years. Generalities and demographics of the patients are shown in Table 2.

### Results of syntheses

All the PROMs improved at last follow-up: KJOC (MD + 25.0; *P* = 0.02), VAS (MD − 5.0; *P* = 0.003), Constant (MD + 40.5; *P* < 0.0001), UCLA-S (MD + 31.2; *P* = 0.006), ASES (MD + 40.0; *P* < 0.0001). Elevation also improved (MD + 22.8; *P* = 0.004). No difference was found in external and internal rotation (*P* = 0.2 and *P* = 0.3, respectively). In this study, only PROMs that were statistically significant were reported. These results are shown in greater detail in Table 3.

**Table 3:** Results of PROMs and ROM

Endpoint	Baseline	Last FU	MD	*P*
KJOC	47.8 ± 11.0	72.8 ± 9.9	25.0	0.02
VAS (0–10)	6.1 ± 0.4	1.2 ± 1.2	− 5.0	0.003
Constant	47.8 ± 19.3	88.2 ± 6.0	40.5	< 0.0001
UCLA-S	18.7 ± 1.8	49.9 ± 16.2	31.2	0.006
ASES	51.3 ± 8.0	91.2 ± 3.4	40.0	< 0.0001
ROM				
Elevation	145.0 ± 4.2	167.8 ± 5.8	22.8	0.004
External rotation	63.0 ± 5.7	70.0 ± 11.3	7.0	0.2
Internal rotation	25.4 ± 20.7	37.9 ± 25.7	12.6	0.3

*FU* follow-up, *MD* mean difference

A total of 75.4% (552 of 692 of patients) were able to return to play within a mean of 6.4 ± 6.0 months, and 62.5% (433 of 692 of patients) were able to return to sport at pre-injury level. Fourteen of 138 patients (10.1%) underwent a further re-operation. Only a few studies reported data on complications, with an overall rate of 7.1% (20 of 280).

## Discussion

According to the main findings of the present study, arthroscopic rotator cuff repair seems to be effective and safe for overhead athletes. The PROMs demonstrated a considerable improvement, with results higher than their minimally clinically important difference (MCID) [23, 43–45]. In total, 75.4% of athletes were able to return to play within a mean of 6.4 ± 6.0 months, and 62.5% of them were able to resume sport at pre-injury level. The rate of complication and revision is of concern (7% and 10%, respectively). However, only a few authors reported the rate of complications. We hypothesised that some authors did not state clearly whether complications were experienced, underestimating this issue.

Among the postoperative complications described in the population studied, the most common complication was re-rupture, and infections the least frequent. Anderson et al. [25] evaluated 48 overhead athletes undergoing arthroscopic rotator cuff reconstruction, reporting 9 (17%) re-tears. The authors found no difference in demographics or functional scores of the shoulders in patients with and without re-tears [25]. Re-tears were evaluated using ultrasound and classified as smaller, the same, or larger than the initial lesion [25]. The authors found no difference in age, ROM, functional scores, or return to sport according to the size of the initial lesion and of the re-tears [25]. However, overhead athletes without re-tears had greater strength in extension and external rotation than those with rupture when evaluated with a portable dynamometer [25]. Liem et al. found a rate of re-tears of 23.8% (5 of 21 operated patients) [4]. However, these athletes showed no difference in sports activity level compared with the group without re-tears [4]. Nevertheless, despite the similarity in activity level, worse functional scores were reported by overhead athletes with signs of re-tear at MRI [4]. The main causes of revision were a trauma after returning to sports, residual pain during overhead activities and stiffness [7, 27, 28].

In the present study, 26.4% (183 of 692 patients) were women, indicating that males could be more prone to rotator cuff injury requiring arthroscopic repair. Young et al. [41] evaluated the return to play in professional female tennis athletes, with an average follow-up of 39 months. The average time to return to play was worse than the overall rate of return to play seen in other studies in which male athletes were considered [26, 34, 46]. Baseball, tennis, volleyball and handball were among the sports in the articles studied, and were the sports accounting for most rotator cuff injuries. The most commonly used technique in these studies for arthroscopic rotator cuff repair was double row repair [7, 25–27, 30, 35, 36, 38, 46–48]. The single-row technique is recommended for tears smaller than 1 cm [49]. For tears sized 1 to 3 cm, it is unclear whether single- or double-row reconstruction should be performed [32, 49–51]. In the present study, the Western Ontario Rotator Cuff Index score was statistically significantly greater in patients undergoing double-row repair, and all the PROMs considered improved from baseline to last follow-up. Also, shoulder elevation improved significantly from baseline to last follow-up; conversely, external and internal rotations did not change significantly. A recent systematic review including 12 studies (347 athletes) evaluated the return to sport in athletes who underwent arthroscopic rotator cuff reconstruction [17]. Most of the athletes considered were involved in overhead sports [17]. Similarly, arthroscopic rotator cuff repair is effective in restoring shoulder function and pre-injury activity level [17]. Another recent systematic review investigated the return to sport in athletes with isolated SLAP (superior labrum anterior to posterior) lesion, including 15 clinical studies (501 athletes) [52]. At an average follow-up of 4 years, up to 87% of patients were able to return to sport [52].

The present systematic review has some limitations. The overall poor quality of the included studies represents an important issue. The CMS average score was 64, with 7 articles not exceeding the individual CMS score of 65 (interpreted as fair quality). The retrospective nature of most of the included studies is an important limitation, which increases the risk of selection bias. The relatively small sample size of most of the included studies also represents another important limitation. The limited average length of the follow-up of the present study, along with the reduced number of procedures included for analysis, may jeopardise the capability to detected uncommon complications. Several heterogeneities between the studies were evident. In general, patients undergo arthroscopic repair of the rotator cuff for persistent instability and/or pain following an acute injury or repeated trauma [7, 29, 32, 53, 54]. However, minimal differences in the surgical indications were found. A further surgical indication in the study conducted by Voos et al. [48] was the presence of a lesion to the labral edge or in the recessed area with apparent laxity and detachment of the insertion of the biceps. In addition, Cohen et al. [29] included patients with unstable injuries of the inferior surface of the rotator cuff, the long head of the biceps tendon, the articular surfaces, and the rest of the labrum. A clinical study [38], on the other hand, included only patients with chronic lesions with full thickness or partial rupture involving more than 50% of the rotator cuff. Most authors combined arthroscopy with other interventions [33]. Subacromial decompression and/or bursectomy, distal resection of the clavicle, acromioplasty, tenotomy and tenodesis of the biceps are the interventions most frequently associated with repair of the rotator cuff [7, 26, 33]. Biceps tenodesis and tenotomy are common procedures among the included studies. The number, location, and nature of the injuries were heterogeneous between the included studies. The supraspinatus tendon is often affected in both acute and chronic injuries in the studies considered [7]. A concomitant lesion of the supraspinatus, infraspinatus and/or subscapularis is also common [26]. SLAP lesions were common in the included studies [29, 30, 32, 35, 36, 40, 55]. However, the characterisation of the lesion was not appropriately described by most authors. Morgan’s classification [56] has been used to evaluate the morphology of the SLAP lesion by some authors [7, 35, 36, 38, 42, 55, 57]. The conclusions of the present study must therefore be considered within the limitations of the present study.

## Conclusion

Arthroscopic reconstruction of the rotator cuff in overhead athletes is effective for improving function of the shoulder in overhead athletes, with a return to sport in 75.4% of patients within an average of 6.4 months. A total of 62.5% of patients were able to return to sport at pre-injury level.

## Data Availability

The data underlying this article are available in the article and in its online supplementary material.

## Abbreviations

PROMs: Patient reported outcome measures
PRISMA: Preferred Reporting Items for Systematic Reviews and Meta-Analyses
KJOC: Kerlan-Jobe Orthopaedic Clinic
VAS: Visual analogue scale
UCLA-S: University of California Los Angeles Shoulder score
ASES: American Shoulder and Elbow Surgeons
CMS: Coleman Methodology Score
MD: Mean difference
FU: Follow-up
